# Radiation-Induced Hydrogel for Water Treatment

**DOI:** 10.3390/gels10060375

**Published:** 2024-05-28

**Authors:** SK Nazmul Haque, Md Murshed Bhuyan, Jae-Ho Jeong

**Affiliations:** Research Center for Green Energy Systems, Department of Mechanical, Smart, and Industrial Engineering (Mechanical Engineering Major), Gachon University, 1342 Seongnam-daero, Sujeong-gu, Seongnam-si 13120, Republic of Korea; sknazmulhaque@gachon.ac.kr

**Keywords:** radiation, water treatment, hydrogel, adsorption, metals and dyes

## Abstract

Along with serving as drug delivery sensors and flexible devices, hydrogels are playing pioneering roles in water purification. Both chemical and radiation methods can produce hydrogels, with the latter method gaining preference for its pure adducts. The water treatment process entails the removal of heavy and toxic metals (above the threshold amount), dyes, and solid wastes from industrial effluents, seawater, and groundwater, as well as sterilization for microorganism destruction. This review analyzed the different types of hydrogels produced by applying various radiations for water treatment. Particularly, we examined the hydrogels created through the application of varying levels of gamma and electron beam radiation from the electron gun and Co-60 sources. Moreover, we discuss the optimized radiation doses, the compositions (monomers and polymers) of raw materials required for hydrogel preparation, and their performance in water purification. We present and predict the current state and future possibilities of radiation-induced hydrogels. We explain and compare the superiority of one radiation method over other radiation methods (UV-visible, X-ray, microwave, etc.) based on water treatment.

## 1. Introduction

Hydrogels and water purification have a close relationship. The living world survives depending on the water on the planet. The major water resources are the ground, lakes and reservoirs, oceans, rivers, and rain. However, only 1% out of 71% of the water on earth is drinkable, raising the demand for purification to supply a sufficient amount of drinking water [1]. Proper purification processes can provide fresh water, which is essential for living organisms. The contaminants added to water come from industrial effluent, agriculture and household sewage, animal manure, insecticides, mine drainage, oil spillage, disruption of sediments, and radioactive waste [2]. By applying electromagnetic radiation to a polymer/monomer blend solution, we prepare radiation-induced hydrogels, a polymeric network that avoids the use of auxiliary chemicals such as initiators and crosslinking agents. They are usually pure materials that can be tuned to obtain the desired composition and stimuli-responsiveness [3]. Water treatment [4], dyes and metal adsorption [5], drug delivery [6], actuators and sensors [7,8], biomedical engineering [9], and energy storage [10] all use radiation-induced hydrogels extensively. Purification is a pressing issue worldwide due to the various sources of pollutants that impact livestock. Above the threshold amount, the pollutants in water are detrimental to living organisms as well as the environment. Skin diseases, diarrhea, and cancer affect human health [11], while turbidity, a collapse in biological oxygen demand, and an increase in total dissolved solids leading to death affect microorganisms and aquatic life [12]. There are many ways to purify the water to make it acceptable to each sector, like drinking, irrigation, and industrial uses. Hydrogels are innovative candidates for purifying contaminated water or seawater. There are different types of hydrogels depending on the preparation technique, and radiation-induced hydrogels receive attention because of their purity, simple manufacturing process, and easy experimental procedure. The radiation process includes gamma, microwaves, electron beams, and UV-visible light [13,14]. Since gamma radiation possesses a frequency of higher energy (>50 keV energy, <0.25 Å wavelength, >12 EHz (1 EHz = 1018 Hz), ionizing ray expressed in gray unit (1 Gy (100 rad) = 1 J/kg), and electron beam as well, which can easily affect the polymers and monomers to undergo free radical formation and polymerization, it takes priority over other radiation techniques having lower efficiency of hydrogel production [15,16,17]. Microwave radiation gives off both heat and electromagnetic radiation at the same time. This makes it more useful for studying (i) step growth [18], (ii) ring opening [19], radical polymerizations [20], and making hydrogels [21]. Shital et al. made a magnetite chitosan-modified polymer composite hydrogel to dispose of dangerous chemicals in the solution, including Chicago sky blue (66.66 mg/g) and Crystal violet dyes (161.81 mg/g). They used a reaction initiator and crosslinking agent to synthesize the polymer under microwave radiation [22]. High temperatures are caused by microwave radiation, which can break down polymers in water or non-water solutions. This can lower the gel content and contaminate the finished products. Furthermore, the lower energy is never enough to activate all types of raw materials for hydrogel production [23]. Among gamma rays, electron beams, and microwave radiation, UV-visible radiation is the easiest and least expensive way to make hydrogels from vinylic monomers [24]. Kunhao et al. reported nanocomposite, tough hydrogels made of TiO_2_, Acrylamide, and *N, N-*dimethylacrylamide that can absorb heavy metals and break down dyes. During UV irradiation upon the blend solution, we used a peroxodisulfate initiator and a *N*, *N*, *N′*, *N′* tetramethylethylenediamine crosslinking agent [25]. However, this technique’s limited use is due to its lower energy frequency, limited passing through the matter, and weak crosslinking [26]. The electron beam-assisted hydrogels show improved mechanical strength and optical transmittance compared to other radiation methods, such as UV-visible [27]. Elena et al. created hydrogels from acrylamide and acrylic acid in water solutions. They used an electron beam irradiator, a trimethylolpropane trimethacrylate crosslinking agent, and potassium persulfate as an initiator. The hydrogels show excellent adsorption of Cu^2+^ and Cr^6+^ [28]. The electron beam can interact with the beam-sensitive polymers and degrade the compound, limiting its use to prepare hydrogels [29]. To prepare biodegradable and efficient hydrogel, natural polymers like pectin, dextrin, starch cellulose and chitosan are widely used in radiation techniques [30,31]. The present study includes a literature review and analysis of hydrogels prepared from different polymers and monomers by using different radiations, their applications, and current prospects.

## 2. Hydrogels and Their Properties

**Figure 1:**
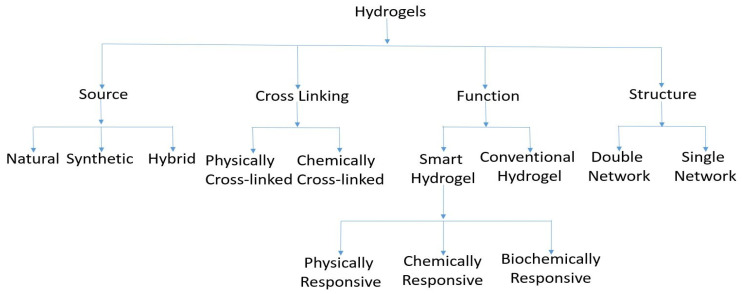
The categorization of hydrogels.

Three-dimensional networks prepared through chemical or physical crosslinking or grafting between polymers or polymers and monomers that can retain a large amount of solvent (water) are categorized as polymeric hydrogels. These types of networks hold functional groups like -COOH, -NH_2_, -NR_2_, -OH, -SO_3_, etc., that are responsible for hydrophilic nature. The swelling makes a hydrogel to extend the void spaces inside the network, followed by the penetration of foreign particles (metals, dyes) and thus the purification of water [32]. Figure 1 represents hydrogel categorization based on its shape, function, structure, morphology, composition, and preparation methods. This phenomenon allows us to classify hydrogel as either natural or synthetic [33]. Based on the charge, we can also classify it into three groups: anionic, catatonic, and non-ionic. The synthesis method divides hydrogen into chemical and physical hydrogels. The physical hydrogel contains some physical activity, effects, and hydrogen bonding, while the chemical reaction accumulates the chemical. Hydrogel’s 3D structure allows for its division into two groups: double and single networks. On the other hand, the double-network hydrogel exhibits higher stability due to its inclusion of two polymers with physically conflicting properties [34]. The higher stability and power are achieved by devising two networks bit by bit [35]. The first-layer network was able to taste hydrogen bonding established by heating and cooling carrageenan a long time ago [36]. We then chemically crosslinked the second-layer network of polyacrylic acid to create a high-strength, self-recovering hydrogel. The kappa carrageenan/polyacrylamide double network hydrogel seems to have good mechanical qualities based on tensile and compression tests. We can categorize hydrogels as either intelligent or conventional based on their functionalities. Intelligent hydrogels, also known as responsive hydrogels, differ from ordinary hydrogels in that they can adapt to external environmental stimuli by changing their morphology, network structure, and mechanical strength [37].

For water purification, hydrogels should possess the following properties: stimuli-responsivity (pH, temperature, etc.), swelling, porosity and permeation, reusability, and biocompatibility. Hydrogels are aquaphilic, naturalistic polymeric chains that effectively capture water in biological fluids due to their water content, porous nature, and easy flexibility. They closely mimic natural living tissues compared to other biomaterials [38]. Hydrogels have two possible chemical durability: they can dissolve and disintegrate over time [39]. Hydrogels are also known as reversible and physical gels. Ionic, hydrogen bonding, or hydrophobic forces initiate a fundamental role, known as molecular entanglement or secondary force, to form the chain. Physical gels, which are generally revocable by dissolving them, change the environmental conditions, such as pH, temperature, and ionic strength [40]. Crosslinking polymers in the dry state or solution can achieve the coupling of covalent bonds connecting separate macromolecular chains in “permanent” or “chemical” gels. Considering the functional group alive in their structure, those gels can be both charged and non-charged particles. By the variation of pH, the charged particle acts as a change in swelling and when it undergoes shape change by the effect of electric field [41]. Owing to the furtherance of the realistic design of the noble gel system, the fundamental gel properties are not so far suitable. It is compulsory to know how the solute molecule relates to the gel for design, especially the partition form between the gel phase and the surroundings phase, and the separation thoroughly depends on the exclusion and molecular attraction effect.

To allow free diffusion of a few solute molecules the adsorbed liquid plays like a particular filter meanwhile, the polymer network performs to grip the liquid jointly. Hydrogels have the capability to absorb water up to thousands of times their dry weight with a lower limit of 10–20%. The type of water present in a hydrogel can determine whether or not nutrients absolutely permeate the gel and discharge biological products. The most polar hydrophilic group in a dry hydrogel will be hydrated by the first water molecule that enters the matrix, resulting in primary bounding water. Furthermore, the appraisal of swelling in the important assay, which can be carried out on hydrogen samples, to the amplitude of the properties. Finding the swelling and swollen state constancy of various gels is a quick, affordable, and reliable method of telling crosslinked gels apart from the original non-crosslinked polymers [42]. Depending on the material’s nature, mechanical properties can change. Higher stiffness is possible by raising the crosslinking degree or decreasing it by applying heat to the materials and diversifying in mechanical properties, which connect to an extensive diversity of variables. For example, crosslinking causes white gelatin to exhibit a noticeable rise in Young modulus [43].

Phase separation and synthesis can create pores in the hydrogels, or they may appear as more compact holes in the network. The specification known as tortuosity compiles several crucial parameters for the hydrogel matrix, including the average pore size, the pore size distribution, and the pore concentration, all of which are challenging to calculate. The film thickness multiplied by the ratio of the pore volume fraction divided by the tortuosity determines the adequate diffusion route length across a hydrogel film barrier. The composition and the crosslink density of the hydrogel polymeric network, in turn, have the greatest influence on these factors. To examine the pore diameters in the hydrogel, labeled molecular probes with a variety of molecular weights (MWs) or molecular sizes are employed. As a feature of hydrogel crosslinking cannot be adequately described; rather it is more of a cause of all the other characteristics of the substance. Crosslinking can occur through several methods like heating, UV radiation, or chemical crosslinking using a crosslinker that triggers large-scale collaborative reactions, including the Michaelis–Arbuzov reaction, nucleophile addition, and so forth [44]. It is achievable to modify a material’s properties and optimize it for a variety of uses by controlling the degree of crosslinking; in this way, a broad range of uses begins with the same basic polymer [45]. Hydrogel is useful in the biomedical industry due to its characteristics of being both biocompatible and non-toxic. Cytotoxicity and in-vivo toxicity tests should undergo most of the polymer. The capacity of a material to work in a distinct application with the right anchor reaction is known as biocompatibility. The bio-safety and bio-functionality parameters comprise biocompatibility [46].

## 3. Radiation-Induced Hydrogels and Their Applications

**Figure 2:**
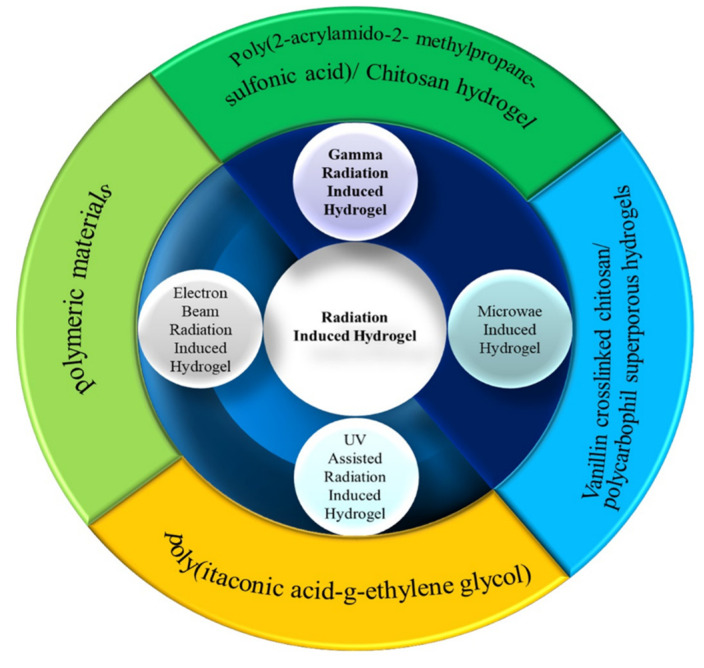
Classification of hydrogel based on applied radiation.

The hydrogels prepared through the interaction between a specific form or range of electromagnetic radiation and polymers or monomers have vast fields of application. Based on the radiation method used, the hydrogels can be classified as shown in Figure 2:

The radiation method proceeds through the formation of free radicals from solvents (H_2_O), polymer backbones, and monomers. The effect of radiation on raw materials is dependent on the energy and frequency range of the applied dose, as well as the types of polymers, solvents, and monomers [47,48]. We choose the radiation based on the criteria that the hydrogels must meet; for instance, we synthesize highly pure hyaluronic acid/chondroitin sulfate-based hydrogels for biomedical applications using gamma radiation [9]. The other criteria include pH-responsive, temperature-pressure-responsive, magnetic-responsive, electric field-responsive, and perfectly cross-lined or grafted hydrogels. Table 1 lists an example of a few very recent hydrogels prepared by applying different radiations and used in different fields of application. Radiation-induced hydrogels contain comparatively fewer impurities, which make them suited for use in diverse fields like water purification to lower the amount of pollutants. In this purification process, both organic and inorganic materials are eliminated. The choice of raw materials and appropriate radiation techniques might be crucial in producing better adsorbent hydrogels. In this instance, the backbone chain and grafting branch to create the multi-functional polymeric networks can be made of natural polymers (pectin, dextrin, cellulose, chitosan, etc.) and functional monomers (vinyl and amide-containing monomers) [49,50,51,52]. The selective adsorption of metal or dye molecules is a noteworthy characteristic of radiation-induced hydrogels. In water purification columns, the anionic and cationic hydrogels can also be employed as ion-exchange resins. Rosiak explained the radiation-induced polymerization in an aqueous solution of polymers and monomers [53].

For example, natural polymer pectin undergoes hydrogel formation with acrylamide upon gamma irradiation, which is briefly described below:

Step 1—Chain Initiation: Upon irradiation, water molecules mainly form three reactive species: hydrated electrons, hydroxyl free radicals, and hydrogen (H_2_), where electrons show very low reactivity toward the formation of polymers. Two hydrogen-free radicals come together to form hydrogen. Hydroxyl free radicals strike the polymer backbone to produce free radical points on that polymer; for example, starch or pectin form free radical points on their chains [54]. At the same time, the vinylic monomer may undergo free radical formation and cyclization [55].

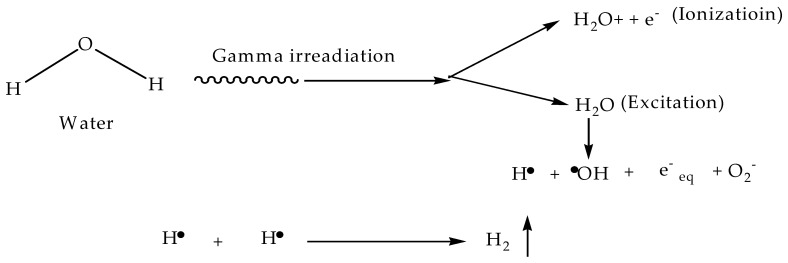

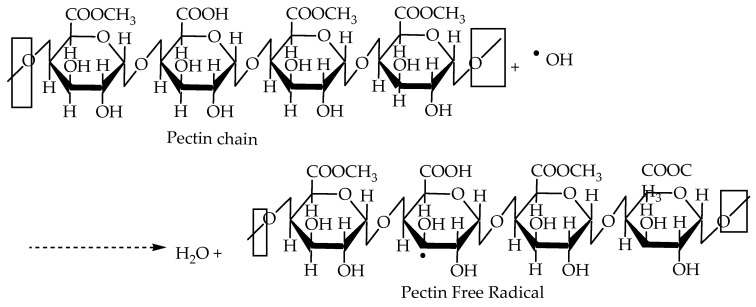





Step 2—Chain Propagation: A macromolecule is formed through the addition of initiating radicals.

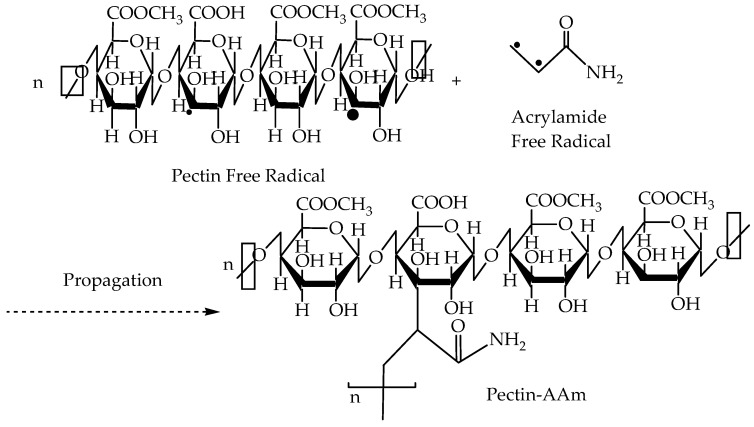



Step 3—Chain Termination: The monomeric and polymeric free radicals are active and unstable tends to undergo final products polymer/hydrogel formation through crosslinking/grafting [56].

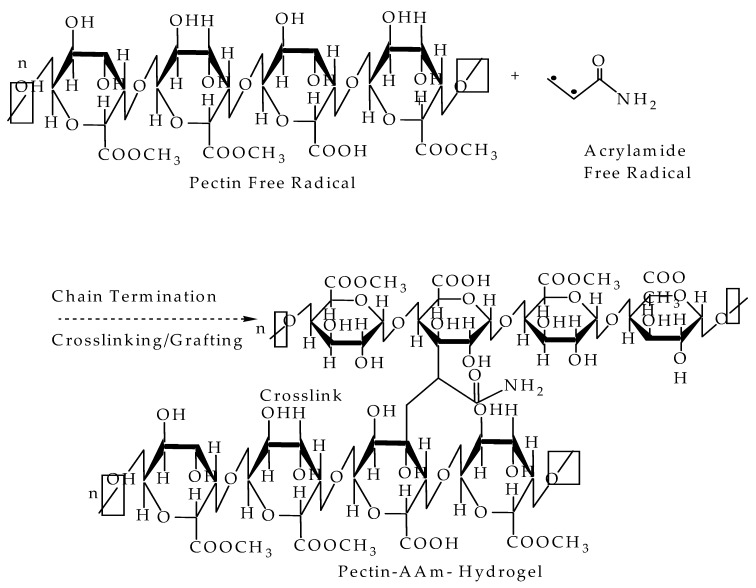



**Table 1:** Some examples of radiation induced hydrogels and their application in water purification and other fields.

S.N.	Hydrogel	Types of Applied Radiation	Application Field of Hydrogel	Reference
**1**	Lyophilized carboxymethyl chitosan hydrogel	Electron Beam	Clinical application	[57]
**2**	1-alkyl −3-vinyl- imidazolium bromide functionalized Tragacanth Gum hydrogel	Electron Beam	Adsorption	[58]
**3**	Lithium Acetate/gelatin/polyacrylamide conductive hydrogel	Gamma Radiation	Flexible strain sensor	[59]
**4**	Poly(vinyl-alcohol)/zirconium NPs/europium hydrogel	Gamma Radiation	-	[60]
**5**	Poly (butyl acrylate) Ethylene vinyl acetate hydrogel	Gamma Radiation	Crude oil flowability	[61]
**6**	Acrylonitrile/methacrylic acid grafted nonwoven fibers hydrogel	Gamma Radiation	Metal recovery	[62]
**7**	Magnetite chitosan-modified polymer composite hydrogel	Microwave Radiation	Pollutant adsorption	[22]
**8**	Alma grafted Methacrylic acid hydrogel	Microwave Radiation	Antimicrobial study	[63]
**9**	Opuntia-carrageenan superporous hydrogel	Microwave Radiation	Drug release and tissue scaffold	[64]
**10**	Poly (ethylene oxide)- poly (ethylene glycol) diacrylate hydrogel	UV-visible Radiation	To prepare CO_2_ selective membranes	[65]
**11**	Ag-Zn nanoparticles (NPs) hydrogel	UV-visible Radiation	H_2_S sensing	[66]
**12**	2-hydroxyethylmethacrilate (HEMA) PCLX-Crosslink hydrogels	Microwave Radiation	Tissue engineering scaffold	[67]
**13**	NiFe_2_O_4_/SANCH or Green superabsorbent nan composite hydrogels	Ultra sound	Metal and dye removal	[68]
**14**	Novel composite hydrogel of poly (HEA/NMMA)-CuS)	Visible Light Irradiation	Sulfamethoxazole(SMX) removal	[69]
**15**	Poly (HEA-co-HAM)-CdS) novel composite hydrogel	Visible Light Irradiation	Bisphenol A (BPA) Removal	[70]

## 4. Water Purification/Treatment

**Figure 3:**
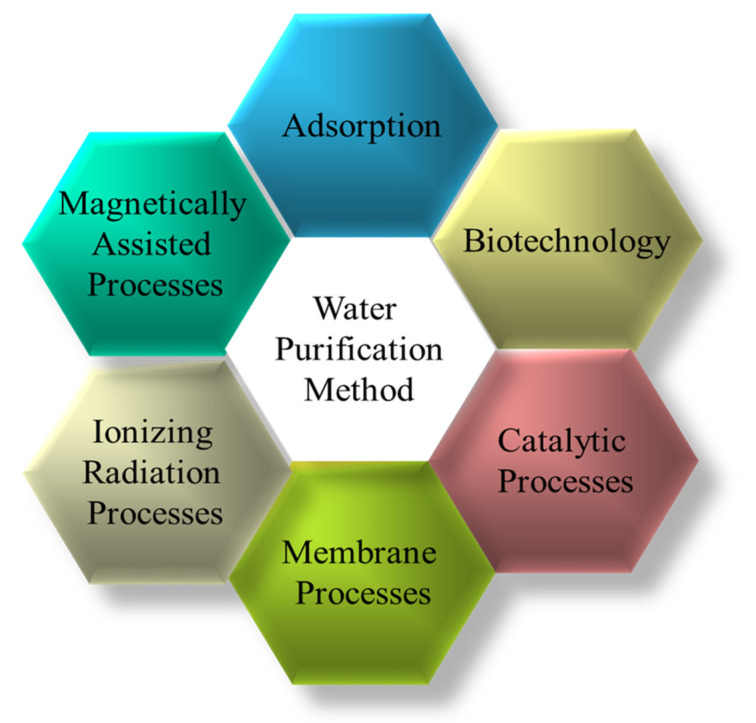
Different water purification methods.

There are few essential elements for an organism to be alive in the environment, and water is one of them. In the modern era, we are producing various consumable products, such as agricultural pesticides, industrial waste, and chemical waste, which directly deteriorate the quality of our drinking water sources [71]. These harmful wastes can lead to various detrimental effects on human health, such as disability, illness, or disorder [72]. Pathogenic microorganisms dominate these wastes, causing water to become tainted. Worldwide, water bodies allow about 2 million tons of impurities from crops, land, factories, and drainage waste to escape, resulting in approximately 1400 deaths per day [73]. The WHO’s recent report predicts that by 2025, 50% of the world’s population will experience a pure water crisis [74]. Furthermore, global climate change could exacerbate the unequal distribution of drinking water and exacerbate water scarcity issues. However, the harmful substances in the tainted water must be decontaminated before use. Water purification is eliminating harmful substances such as chemicals, solids, biological toxins, and gases from the water to make it suitable for the intended purpose [71]. Hence, the evolution of a methodical, inexpensive, environmentally safe, and adaptable technology is very significant to water purification [73]. The limitations of using different micro-particles to eliminate contrasting contaminants vary, such as the time it takes for the micro-particles to reach the finite surface area for the transport capability of the toxic substances [5]. Likewise, for the 3D graphene-based macrostructures for water treatment, the low-dimensional nanomaterial’s restraining forces correlate with its small size. First, it forms clusters in water and loses a lot of its activity. Second, it is hard to return to the nanomaterials drained from the water being studied, and it costs a lot to perform a membrane-based dissociation process. Furthermore, it also encounters environmental and health issues. However, photocatalytic advanced oxidation processes for water treatment have some problems, such as low eradication efficiency, high costs, a lot of work, too much dirt output, being good for limited improvement, not being suitable for wide use, and making some harmful substances [75]. Figure 3 presents the various methods mostly used for water treatment where the ionizing radiation processes are receiving priority. Table 2 lists the worthy materials used to purify contaminated water.

## 5. Radiation-Induced Hydrogels for Water Treatment

Grafting or crosslinking radiation-induced hydrogels is a valuable technique for modifying biopolymers for various applications, particularly in the water purification field. We use various hydrogels to purify water with required parameters above the threshold amount. For specific contaminants like metal ions, dyes, or trace amounts of anions, we can use a specific hydrogel. We are living in a modern era where the industrial revolution plays a significant role in every aspect of human beings. The development of industry has had a significant impact on the environment and its resources, such as water, which are essential for human survival. Moreover, overexploitation, global warming, unequal distribution, and human growth also play a principal role in this calamity [85]. As a result, make use of contaminated water, sewerage water, materializing contaminants (dyes, minerals, decomposable waste, agriculture pesticides and manure, poisonous pollutant pharmaceutical waste), and cosmetics [86]. Radiation-induced hydrogels are more advantageous because they have enhanced swelling properties, tunable properties, improved efficiency, sterile production, and versatility, and are environmentally friendly. For microwave-assisted hydrogel synthesis, adding polymerization automatically requires less reaction time and side reactions, whereas it cannot be used to make cast hydrogel from monopolymers [87]. Magnetite-chitosan-modified polymer composite hydrogel has insufficient performance for dye eradication [22]. Table 3 displays the water quality parameters and the hydrogels that the researchers are currently using to control the quantity if they exceed the threshold amount.

## 6. Purification of Water by Removing Toxic and Heavy Metals

Humanity is constantly searching for a healthy source of food and drink free of industrial pollution and toxic heavy metals that damage public health in order to improve people’s quality of life in the face of disease, aging, and death [92]. We can further optimize the selectivity and efficiency of radiation-induced hydrogels, building on their impressive capabilities in water purification. Living creatures easily accumulate heavy metal ions due to their high toxicity, great permeability, and persistent accumulation. This can have long-term negative effects on humans and other species [97]. The hydrogel network’s functional groups significantly contribute to the adsorption process, especially at acidic pH levels, where their activity intensifies. One of the most important parts of water purification is the removal of toxic and heavy metals from wastewater, seawater, and groundwater for use in a variety of industries. These metals include lithium, beryllium, aluminum, sodium, potassium, cobalt, iron, chromium (III, IV), zinc, copper, arsenic, cesium, etc. Therefore, more research is required to eliminate and identify these contaminants. Significant metals are present in water, a crucial element for a secure and robust environment on Earth [81,98]. According to the US EPA’s cumulative data for the unsafe material described [99], certain metal ions, such as Cr (VI), As (V), Hg (II), Cu (II), and Pb (II), are highly poisonous and non-biodegradable [100,101]. Lead and mercury are the most widely accepted and extensively considered metals that are eminently poisonous to the human brain and nervous system. The numerous commercial uses of highly lethal, needless metals like lead and mercury contribute to the advancement of poison pollution in the surrounding areas [102]. Industries frequently employ compounds containing mercury and lead in a variety of settings. Mercury oxide, the raw material for mercury batteries, often undergoes breakdown to yield primary mercury. Any kind of food can consume or suck in the highly fatal mercury admixture, and sea fish such as tuna and shark have been known to consume a portion of lead [103]. Humans can come into contact with mercury in a variety of ways, such as through tainted food, the battery industry, or dental amalgam [104]. Lead, an extremely dangerous substantial metal, is the primary cause of harm to plants’ photosynthetic processes and lipid membranes [105]. A wide range of sectors utilize lead, including battery recycling, lead smelting and processing, pigment, solder, plastics, cable sheathing, ammunition, and ceramics [106,107]. Removing these substances, especially from water, is imperative to reduce the hazards. Researchers have used a variety of investigations, applications, and techniques to remove these components. These include membrane filtration, ion exchange, irradiation, chemical precipitation, coagulation, flotation, reverse osmosis, electrochemical approaches, and adsorption [108,109].

**Figure 4:**
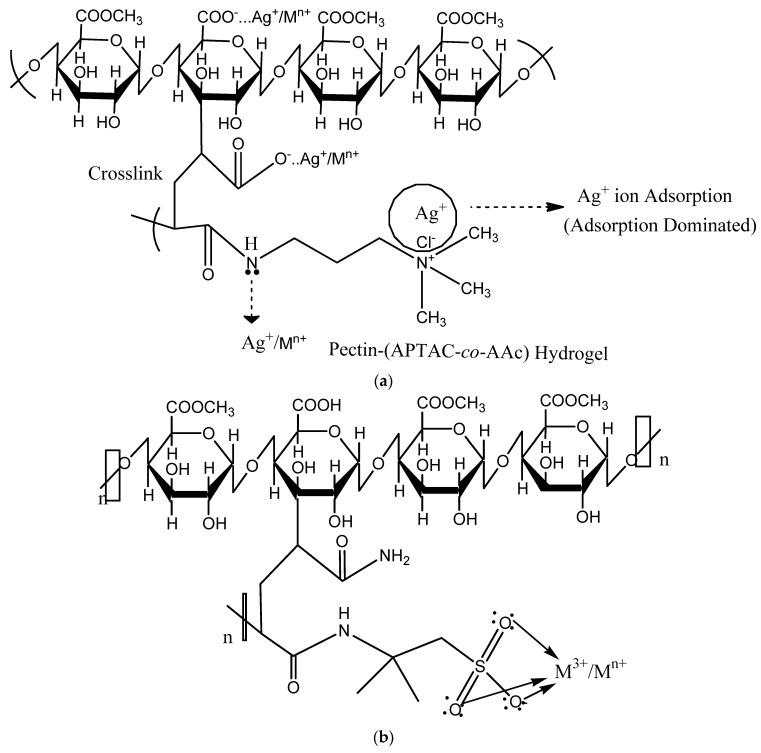
Metal adsorption mechanism on (**a**) Pectin-(APTAC – co - AAc) and (**b**) Pectin - AAm -AMPS hydrogels prepared by gamma radiation, replotted with permission.

Radiation-induced hydrogels exhibit excellent metal ion adsorption in both single-metal and multi-element solutions. A few hydrogels exhibit selective adsorption in multi-element solutions. There was work we did where we used gamma radiation to make pectin-[(3-acrylamidopropyl) trimethylammonium chloride-co-acrylic acid] [110] and pectin-acrylamide-(2-Acrylamido-2-methyl-1-propanesulfonic acid) [90] hydrogels. We then used them to selectively adsorb silver (Ag^+^) and trivalent metal ions (Al^3+^, Cr^3+^, Fe^3+^, Ga^3+^, and In^3+^) from a solution with 27 metal elements. Metal size, charge, and standard electrode potential were the factors affecting selectivity and adsorptive efficiency. Figure 4 illustrates the absorption of metal ions at pH levels below 3. The hydrogel network’s functional group, highly activated at this pH level, captures metal ions through their negative charges. The metal ions compete among themselves based on their ionic size and standard electrode potential values. In Figure 4a, silver metal has a high affinity for the chloride ion, which facilitates being adsorbed first [111,112]. Silver ions occupy the adsorption sites, allowing other metals to adsorb. These hydrogels enable the removal of silver metal from both seawater and groundwater. Figure 4b demonstrates the hydrogels’ greater attraction to trivalent metal ions. This is because their structure contains a sulfonic group that attracts metal ions based on their ionic charges. Trivalent metals compete with each other and have adsorption sites. The trivalent metals also compete within themselves via their respective ionic sizes [113].

## 7. Purification of Water by Removing Dyes

**Figure 5:**
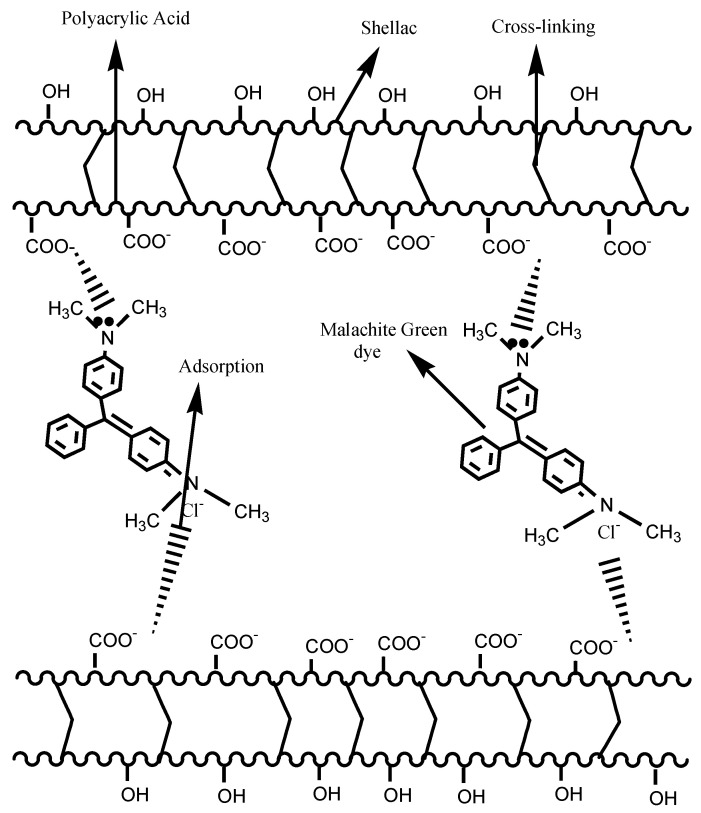
Probable mechanism of cationic malachite green dye adsorption by radiation-induced polyacrylic acid/shellac hydrogels [116].

Industrial effluent contains various types of dye contaminants that have detrimental effects on living organisms and the environment. Hydrogel is an efficient way to dispose of any kind of color, neutral, negative, and positive. Most of the time, we use the cationic hydrogel (ex-quaternary ammonium chloride-containing) for dyes that are negatively charged, like sodium 4-(2-hydroxy-1-naphthylazo) benzenesulfonate, and the anionic hydrogel poly (methacrylic acid-co-methacryloxyethyl glucoside) [114] for dyes that are positively charged, like methylene blue [115]. Figure 5 shows how gamma-radiation-induced novel superabsorbent polyacrylic acid/shellac hydrogel is used to remove malachite green (MG) dyes from a water solution. The nitrogen atoms localize two amine groups of MG, a cationic dye, to form positive single charges. Physical adsorption resulted in monolayer formation, followed by multilayer formation. Here, we assessed the two most likely pathways for dye adsorption. One had to do with how the negatively charged adsorption sites (-OH, -CO, -COO−, and -COOH) on the polymer interacted with the dye that was adsorbent. These sites were hydrophilic in different ways. The second method used hydrogen bonds between the NR_2_ amine groups in the basic dye and the OH or -COOH functional groups on the surface of the polymer. The addition of SH caused a crosslinking process, increasing the number of ionizable PAA groups. This resulted in the formation of PAA/SH hydrogels with significantly more carboxyl groups than PAA. This may strengthen the bond between the dye’s cationic groups and the anionic groups on the surface of the resulting polymer blends. -OH, -CO, -COO^−^, and -COOH are groups of negatively charged ions that are on the surfaces of the PAA and PAA/SH hydrogel. These ions will interact with the positively charged MG dye molecules and help the MG dye stick to them. Nonetheless, it seemed that the primary intermolecular interaction in this technique was the electrostatic connection between the dye molecules and the hydrogel matrix [116].

**Figure 6:**
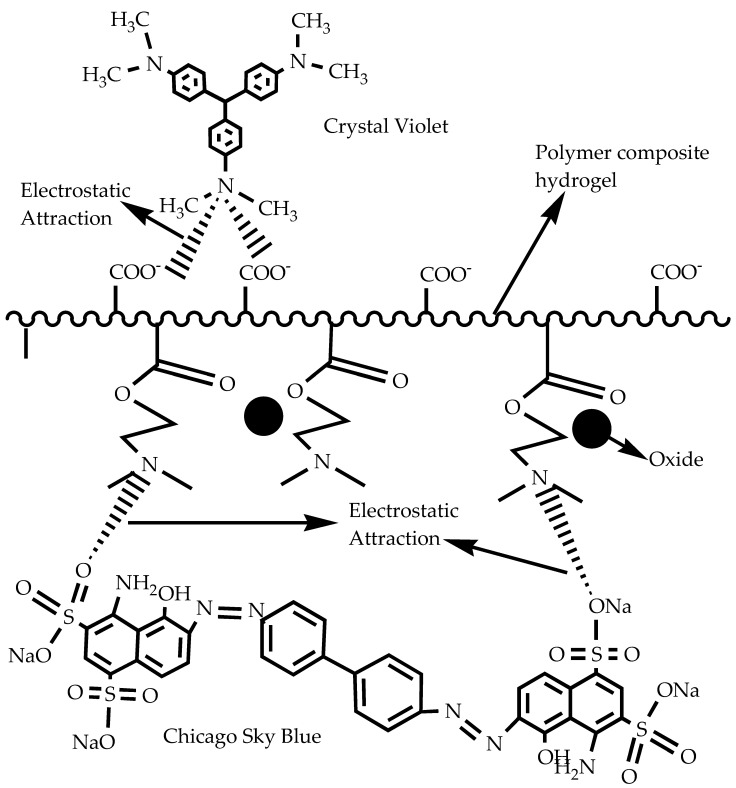
Probable rapid dye adsorption mechanism by magnetite chitosan-modified polymer composite hydrogel.

Heat-treated chitosan-grafted (CA-co-DMAEMA)/Fe_2_O_3_ hydrogels can absorb both cationic (Crystal Violet, CV) and anionic (Chicago Sky Blue, CSB) dyes from water or solutions. Both dyes are carcinogenic when discharged into the environmental system. Fe_2_O_3_ nanoparticles enhance the magnetic properties of the hydrogels, facilitating their easy separation following dye absorption. Figure 6 illustrates the likely absorption process of CV and CSB dyes. The sponge-like structure and many functional groups in the hydrogel make it easier for the dyes to stick and absorb. The hydrogel can interact with many different types of dye molecules because it is amphiphilic, which means it has both hydrophilic (which attracts water) and hydrophobic (which repels water) parts. Functional groups in the hydrogel, such as amine and carboxyl groups, connect with the dyes through the porous structure shown in Figure 6. This dual mechanism effectively removes both CV and CSB from the solution, thereby significantly reducing the risk of environmental contamination. The hydrogel’s design not only captures the dyes but also allows for regeneration and reuse, making it a sustainable solution for wastewater treatment. The integration of such advanced materials into environmental management strategies holds great promise for the future of water purification technologies [22].

## 8. Advantages and Limitations of Radiation-Induced Hydrogel for Water Treatment

In the realm of hydrogel synthesis, the choice of radiation technique is pivotal for determining the quality and applicability of the final product. The radiation techniques have advantages over other methods, such as the solution method for hydrogel preparation. Of the four radiation techniques, UV and microwave radiation are the least energetic and produce heat, which limits their use in hydrogel production. On the other hand, gamma rays and electron beams stand out as the more potent options, widely recognized for their efficacy in initiating polymerization reactions necessary for hydrogel formation. Only a very limited number of hydrogel preparations contain UV-visible electromagnetic radiation. Both microwaves and UV rays weaken the chemical and mechanical bonds between hydrogels, indicating the need for fewer repetitions in the water purification field. Despite their popularity, gamma rays and electron beams are not without their challenges. Presently, gamma rays and electron beams are widely acceptable and used for hydrogel synthesis, followed by water purification. The major problem with the two radiations is that they degrade raw materials during irradiation for polymerization reactions. The electron beam is the best for a fast rate of gel formation, while for a slow rate, the gamma ray is suitable. However, gamma radiation’s ability to penetrate materials where the electron beam has a lower penetration limit sets it apart from the other two radiations. The higher initial experimental setup is another disadvantage of both processes. Furthermore, the environmental impact of these radiation techniques is a consideration that cannot be overlooked. The disposal of radioactive sources and the management of irradiated materials pose challenges that must be addressed to ensure sustainable practices in hydrogel production [49]. The disadvantages are specifically for the raw materials, which have no vinylic groups and do not undergo solution with water solvents.

In conclusion, while gamma rays and electron beams are currently the preferred methods for hydrogel synthesis, ongoing research and development are essential to overcome the limitations associated with these techniques [117]. Discoveries in radiation technology and materials science could lead to the creation of new methods or improvements to old ones. This would make the process of making hydrogels for cleaning water and other uses more effective and long-lasting [118].

## 9. Future Prospects and Conclusions

Later research can focus on making these hydrogels more selective by adding more functional groups that target specific pollutants like metals, dyes, and other substances, or by changing the network topology to change how easy it is for pollutants to attach. This might result in even more effective purification systems that can remove dangerous pollutants from complicated aqueous solutions with selectivity, enhancing the water’s safety and use in a variety of applications.

In summary, radiation-induced hydrogels have emerged as a transformative solution for water purification, addressing the critical need for clean water in various environments. Radiation triggers the polymerization of monomers, synthesizing these hydrogels into a three-dimensional network that can absorb and retain large quantities of water. The unique structure of these hydrogels allows for the selective removal of contaminants, including toxic heavy metals and dyes, which are often present in industrial effluents and pose significant environmental and health risks. We use different hydrogels to remove different pollutants from their respective sources, such as seawater, groundwater, and industrial effluents. The wastewater successfully removes toxic and heavy metals and dyes. Renowned research articles review the hydrogel preparation methods and the irradiation-induced polymerization process. We also explained the mechanisms of metal and dye adsorption. The development of radiation-induced hydrogels represents a significant leap forward in water purification technology. With their ability to efficiently remove a wide range of pollutants and their adaptability to various environmental conditions, these hydrogels hold the promise of providing clean, safe water to communities worldwide, thus contributing to the global effort to ensure access to clean water for all. Finally, it can be concluded that radiation-induced hydrogels are promising materials for water purification.

## Figures and Tables

**Table 2:** The materials used in water purification.

S.N.	Materials	Species Removed	Types of Water Purified	Efficiency	Reference
**1**	Wheat straw inoculated with microalgae	Contaminants, including nutrients and heavy metals.	Ground water	Removed most of the polluting components from groundwater.	[71]
**2**	Hydrogels	Heavy metals, Dyes and solid wastes.	Wastewater, ground water, Sea water	Remove most of the pollutants	[76]
**3**	Activated charcoal	Methylene blue degradation and the heavy elements	Drinking water	96% removal of heavy metal and the Methylene Blue removal almost 94%.	[77]
**4**	Water Hyacinth	Heavy Metals	Sewage waste water	65% removal	[78]
**5**	Coco peat	Organic solid	Wastewater	45% reduction	[79]
**6**	Rice husk	NaCl	Groundwater, Seawater	adsorption of 27.83%,	[80]
**7**	Ion exchange resin	Reducing hardness of water	Hardwater	71% removal	[81]
**8**	Magnetic graphene oxide	Toxic heavy metal ions (Pb^+2^, Cr^+3^, Cu^+2^, Zn^+2^ and Ni^+2^)	Drinking water	89.612% for Pb^+2^ at pH 5, 92.033% for Cr^+3^ at pH 6, 92.433% for Cu^+2^ at pH 6, 90.383% for Zn^+2^ at pH 7 and 92.233% for Ni^+2^ at pH 8	[82], [82]
**9**	Multiwall carbon nanotube–zirconia Nano-hybrid	Adsorption of arsenic	Drinking water	92% As(III) and 95% As(V), respectively	[83]
**10**	Multifunctional porous β-cyclodextrin polymer	COD	Natural water treatment	92% removal	[84]

**Table 3:** A few water quality parameters [88] and radiation-induced hydrogel used for the extra amount.

S.N.	Water Quality Parameters	Threshold Limit	Unit	Hydrogel Used for Purification	Reference
**1**	Arsenic	0.05	mg/L	Polyacrylamide-Carboxymethylcellulose hydrogels	[89]
**2**	Chromium	0.05	mg/L	Pectin-acrylamide-(2-Acrylamido-2-methyl-1-propanesulfonic acid) hydrogels	[90]
**3**	Lead	0.05	mg/L	Polysaccharides–polyvinyl alcohol hydrogels	[91]
**4**	Mercury	0.002	mg/L	Wheat Flour/Acrylamide hydrogels	[92]
**5**	Nitrate (as N)	10.00	mg/L	Polyacrylamide carboxymethylcellulose hydrogels	[89]
**6**	Silver	0.05	mg/L	(3-acrylamidopropyl) trimethylammonium chloride-acrylic acid functional superabsorbent hydrogels	[93]
**7**	Copper	1	mg/L	Polyvinyl alcohol/acrylic acid polymeric hydrogels	[94]
**8**	Iron	0.3	mg/L	Pectin-acrylamide-(2-Acrylamido-2-methyl-1-propanesulfonic acid) hydrogels	[90]
**9**	Manganese	0.05	mg/L	Polysaccharides–polyvinyl alcohol hydrogels	[91]
**10**	Sulfate	250	mg/L	(2-hydroxyethyl methacrylate) - N-vinylpyrrolidone hydrogels	[95]
**11**	Total dissolved solids	500	mg/L	2-hydroxyethyl methacrylate/acrylamidopyridine hydrogels	[96]
**12**	Zinc	5	mg/L	Polyvinyl alcohol/acrylic acid polymeric hydrogels	[94]

## Data Availability

Not applicable.
